# Brain health and the gut microbiome (bMicrobiome Study): a proof-of-concept, feasibility study integrating shotgun metagenomics, metrology, and multidimensional phenotyping across the cognitive aging spectrum

**DOI:** 10.1080/29933935.2026.2679810

**Published:** 2026-06-09

**Authors:** Leigh A. Frame, Alison Warren, Abdelmohsen Al Qalam, Patrick G. Corr, Mina Farah, Michaela Karam, Katherine Rangoussis, Mehrshad Fahim Devin, Zeynep Celikkol, Lindsey Gordon, Daniel Villarreal, Elizabeth Catto, Yvonne Udam, Katherine Thompson, Owen Lubinski, Abdallah Samman, Alia Badawi, Holly Hack, Monique Hunter, Ian Hines, Stephanie Servetas, Scott A. Jackson, Nur A. Hasan, Mikhail Kogan

**Affiliations:** a Frame-Corr Laboratory, Department of Clinical Research and Leadership, George Washington University School of Medicine and Health Sciences, Washington, DC, USA; b Department of Clinical Research and Leadership, George Washington University School of Medicine and Health Sciences, Washington, DC, USA; c Office of Integrative Medicine and Health, George Washington University School of Medicine and Health Sciences, Washington, DC, USA; d Department of Physician Assistant Studies, George Washington University School of Medicine and Health Sciences, Washington, DC, USA; e Recondite Consulting, Saint Michaels, MD, USA; f Biosystems and Biomaterials Division, National Institute of Standards and Technology (NIST), Gaithersburg, MD, USA; g The NEST Consulting, Saint Michaels, MD, USA; h EzBiome, Inc., Gaithersburg, MD, USA; i Arius Bioscience, Inc., Olney, MD, USA; j GW Center for Integrative Medicine, Washington, DC, USA; k Geriatrics & Palliative Medicine, George Washington Medicine, Washington, DC, USA; l Department of Medicine, George Washington University School of Medicine and Health Sciences, Washington, DC, USA

**Keywords:** Gut microbiome, cognitive aging, mild cognitive impairment, Alzheimer's disease, gut–brain axis, shotgun metagenomics, metrology, precision nutrition

## Abstract

**Background:**

Associations between the gut microbiome and cognitive decline remain inconsistent, reflecting methodological variability, small cohorts, and limited integration of behavioral and lifestyle factors. The microbiota–gut–brain axis may influence cognition through metabolic, immune, and neuroendocrine pathways affecting mood, decision-making, and health behaviors.

**Methods:**

This prospective, proof-of-concept study integrated multidimensional phenotyping with metagenomic sequencing (shotgun) in adults (50–90 y) around Washington, DC. Participants were classified as healthy controls (HC) or mild cognitive impairment (MCI) by clinical history; early Alzheimer's disease (eAD) participants were unable to complete study requirements. Longitudinal assessment used Boston Cognitive Assessment (BoCA), patient-reported outcomes (PROMIS-29), dietary intake and quality (DietID™), readiness-for-change (adapted URICA), at-home stool sample collection.

**Results:**

Seventeen participants completed sufficient assessments (HC *n* = 11; MCI *n* = 6). Substantial overlap in gut microbiome composition was observed between HC and MCI. Poorly characterized or uncommon taxa drove trends; unassigned taxa were common. Assessment revealed high diet quality and variability in dietary patterns and key components (vegetables, whole grains, fat, fish). Participants demonstrated high readiness to engage in nutritional behavior change, with individuals with MCI reporting greater concern about maintaining changes and a stronger desire for external support.

**Conclusions:**

Integrating multidimensional phenotyping with metagenomics is feasible in cognitive decline. Findings highlight biological and behavioral heterogeneity, limitations of species-level inference, and diet and behavioral readiness as modifiable contextual factors.

## Introduction

Alzheimer's disease (AD) and related dementias constitute a rapidly escalating global public health challenge, with prevalence projected to increase substantially as populations age worldwide.^1^ ^,^ ^2^ Mild cognitive impairment (MCI), which often precedes dementia, represents a critical window for early identification and intervention, particularly given the limited efficacy of currently available disease-modifying therapies once neurodegeneration is advanced.^3^ ^,^ ^4^ As a result, there is growing urgency to identify modifiable biological, behavioral, and environmental contributors to cognitive decline that may be targeted earlier in the disease course.

### The microbiota–gut–brain axis and cognitive aging

The microbiota–gut–brain axis has emerged as a compelling and biologically plausible pathway linking peripheral physiology to central nervous system function. This bidirectional system integrates microbial metabolites, immune signaling, neuroendocrine pathways, and neural communication via the vagus nerve, collectively influencing neuroinflammation, blood-brain barrier integrity, neurotransmitter synthesis, and synaptic plasticity.^5^ ^,^ ^6^ Disruptions within this axis have been implicated in multiple neuropsychiatric and neurodegenerative conditions, including depression, Parkinson's disease, and Alzheimer's disease.^7-9^ Emerging evidence further suggests that the microbiota–gut–brain axis interacts with neural circuits involved in mood regulation, motivation, and decision-making, processes that may influence health behaviors, treatment adherence, and lifestyle modification relevant to cognitive aging.^9^ ^,^ ^10^

Human studies increasingly report associations between gut microbiome dysbiosis and MCI or AD, including altered alpha and beta diversity, enrichment of pro-inflammatory or pathobiont taxa, and reduced abundance of short-chain fatty acid (SCFA)-producing bacteria such as *Faecalibacterium*, *Roseburia*, and *Eubacterium.* ^11–13^ SCFAs, particularly butyrate, play a key role in maintaining gut barrier function and regulating neuroimmune responses, and their depletion has been hypothesized to contribute to chronic neuroinflammation and cognitive vulnerability.^14^ ^,^ ^15^

### Heterogeneity and limits of the existing human evidence

Although the human literature broadly supports an association between gut dysbiosis and cognitive impairment, the clinical signal remains difficult to interpret. Comparisons across studies remain challenging due to substantial variability in cohort design, sampling protocols, sequencing approaches, and analytic pipelines, limiting reproducibility and translational relevance. In our recent scoping review of human studies examining the gut microbiome in MCI and AD, gut dysbiosis was frequently reported, including shifts in microbial diversity and broad compositional patterns; however, even putatively “recurring” taxa were inconsistently identified across studies and populations.^16^

Methodological heterogeneity emerged as a dominant limitation. Studies varied widely in sampling protocols, sequencing platforms, bioinformatic pipelines, and statistical thresholds, and many reported results only at higher taxonomic levels (e.g. phylum or family), limiting mechanistic inference and cross-study comparability.^9^ ^,^ ^17^ This problem is compounded by ongoing taxonomic reclassification and evolving microbial nomenclature, which can obscure whether ostensibly similar findings reflect the same organisms across studies, further complicating synthesis and interpretation. In this manuscript, the current taxonomy is used throughout and the former taxonomy is noted at first use only.

### Design constraints and translational gaps

A second major limitation identified in the scoping review was the predominance of study designs that constrain causal inference and translational utility. Most included studies were cross-sectional or retrospective case-control designs, precluding conclusions about directionality or temporal dynamics as cognitive impairment progresses.^9^ ^,^ ^18^ Intervention studies (most commonly probiotic or dietary interventions) demonstrated promising signals in select cognitive domains, but results were inconsistent, and trials were generally small, heterogeneous, and of short duration (typically ≤24 weeks), limiting confidence in durability, generalizability, and biomarker discovery.^16^ ^,^ ^19^

Geographic concentration further limits transportability. A substantial proportion of human microbiome-cognition studies have been conducted in China and other East Asian populations, with relatively few studies in U.S. cohorts.^9^ Given well-established population-level variation in gut microbiota driven by diet, lifestyle, environment, and healthcare systems, this imbalance raises concerns about external validity and the applicability of findings across diverse clinical contexts.^16^ ^,^ ^20^

### Stress, immune signaling, and the MGIBA

Importantly, emerging work suggests that microbiome-brain relationships cannot be fully understood without accounting for stress physiology and immune modulation. Chronic psychosocial stress alters gut permeability, immune activation, and microbial composition, amplifying inflammatory signaling along the microbiota–gut–immune–brain axis (MGIBA), including the resident immune cells, microglia.^9^ ^,^ ^21^ ^,^ ^22^ Our prior work has highlighted how sustained activation of the stress response may interact with gut dysbiosis to exacerbate neuroinflammatory processes relevant to cognitive decline and mental health outcomes.^9^ Yet few human microbiome studies in MCI or AD systematically integrate stress, psychosocial factors, or immune markers into their analytic frameworks, representing a critical blind spot in the field.

### Implications for study design: the bMicrobiome Study

These limitations directly informed the design of the Brain Health & the Microbiome (bMicrobiome) Study in three key ways. First, to address temporal uncertainty, the study adopted a prospective, longitudinal framework with repeated biological sampling and repeated cognitive, functional, dietary, and psychosocial assessments, enabling characterization of within-person dynamics over time rather than relying solely on between-group comparisons.

Second, to move beyond descriptive compositional analyses and improve mechanistic interpretability, the study prioritized higher-resolution microbiome characterization using shotgun metagenomic sequencing rather than relying exclusively on 16S rRNA sequencing. This approach allows for functional profiling of microbial pathways, including metabolic and immune-relevant gene systems, which may be more closely linked to cognitive outcomes than taxonomic composition alone.^23^ ^,^ ^24^

Third, recognizing that inconsistent methods and analytic pipelines represent a major barrier to synthesis, the study incorporated metrology-aligned approaches to sampling, sequencing, and data processing to enhance reproducibility and cross-study comparability. Findings are explicitly framed as exploratory and hypothesis-generating, reflecting the current state of the evidence base and aligning analytic claims with appropriate inferential limits. Collectively, this design seeks to advance the field from fragmented associative findings toward more coherent, reproducible, and translationally meaningful insights into the role of the microbiome in cognitive aging.

The primary objective of this study was to assess feasibility and characterize exploratory associations between gut microbiome features and cognitive health across healthy aging, MCI, and early AD.

## Methods

### Study design and oversight

This prospective, longitudinal, observational cohort study enrolled adults aged 50–90 y residing in the Washington, DC metropolitan area, which includes the District of Columbia and parts of Maryland, Virginia, and West Virginia, where commuting into DC is common. Participants were followed at baseline, 3 months, and 6 months. The study protocol was approved by the institutional review board of The George Washington University, and all participants or their legally authorized representatives provided digital informed consent. This study was registered on ClinicalTrials.gov (Identifier: NCT06039267, Table 1).

**Table 1.** Study outcome domains.

Outcome domain	Measure description	Time frame
Gut microbiome composition	To compare the gut microbiomes of patients with early Alzheimer's disease, mild cognitive impairment, and healthy controls using diversity as well as genus, species, and strain level differences in composition (shotgun metagenomics)	3 months and 6 months
Gut microbiome function	To compare the gut microbiomes of patients with early Alzheimer's disease, mild cognitive impairment, and healthy controls using diversity as well as genus, species, and strain level differences in function (shotgun metagenomics)	3 months and 6 months
Document microbiome changes following lifestyle changes for future study design	Observationally collect gut microbiome and lifestyle changes to inform the design of a trial to study lifestyle interventions	3 months and 6 months

### Participants and eligibility

Participants were categorized into three groups: healthy controls, MCI, or early Alzheimer's disease (eAD), based on pre-existing clinical diagnosis and clinical history, and the Brief Cognitive Assessment (BoCA) was administered at baseline to confirm consistency with the reported cognitive category and to support longitudinal monitoring(described below). Inclusion criteria required the ability to complete study visits and provide stool samples. Exclusion criteria included major gastrointestinal disease, recent antibiotic use, or conditions expected to substantially alter gut microbiota independent of cognitive status. Detailed inclusion and exclusion criteria are summarized in Table 2.

**Table 2.** Study inclusion and exclusion criteria.

Category	Criteria
Inclusion criteria	Adults aged 50–90 y Residing in the greater Washington, DC metropolitan area Classified as one of the following: – Early Alzheimer's disease (eAD) – Mild cognitive impairment (MCI) – Healthy control (no diagnosis of eAD or MCI) Able to provide informed consent or have a legally authorized representative provide consent
Exclusion criteria	Presence of medical, neurological, psychiatric, or other conditions that, in the judgment of the study team, could affect study outcomes or alter the risk-benefit ratio
Additional study characteristics	All sexes eligible Non-probability sampling Prospective cohort

Although the original recruitment target was 45 participants (15 per group), withdrawals and exclusions resulted in a final analytic sample of 17 participants. Reasons for attrition included participant burden, illness, and challenges related to stool collection.

### Clinical and behavioral assessments

Clinical, functional, dietary, and psychosocial assessments were selected to capture multidimensional contributors to cognitive health and to align with emerging frameworks emphasizing whole-person phenotyping in neurodegenerative research. Cognitive function was assessed using the BoCA, a brief, validated instrument designed to detect subtle cognitive changes across memory, executive function, attention, visuospatial reasoning, language, and orientation.^25^ The BoCA was administered at each study visit to support longitudinal tracking of cognitive performance across the cognitive aging spectrum. BoCA scores were used to support cognitive phenotyping and group characterization, rather than diagnostic determination.

Participants enrolled in the study had a pre-existing diagnosis of mild cognitive impairment or Alzheimer's disease established prior to enrollment. Diagnoses were confirmed through comprehensive evaluation at the affiliated Memory Clinic or via systematic review of external medical records to ensure that diagnostic criteria had been appropriately met. In cases where diagnostic documentation was incomplete or ambiguity remained, an adjudication process was implemented requiring independent review and consensus agreement by at least two trained memory clinicians, thereby enhancing diagnostic validity and reducing the risk of misclassification.

Functional status was measured with the Alzheimer's Disease Cooperative Study–Activities of Daily Living (ADCS-ADL), which assesses the ability to perform everyday activities relevant to independent living and disease progression.

Psychosocial health was evaluated using the PROMIS-29 profile, capturing key domains including physical function, anxiety, depression, fatigue, sleep disturbance, social participation, and pain interference. These measures were included to contextualize cognitive findings within broader psychosocial and quality-of-life dimensions known to interact with neuroinflammatory and stress-related pathways.

Dietary intake and diet quality were assessed using DietID™, a validated, rapid dietary assessment tool that characterizes habitual dietary patterns relevant to cardiometabolic and inflammatory health using Diet Quality Photo Navigation (DQPN®), a novel pattern-recognition approach requiring no detailed recall or logging of foods.^26-30^ DietID presents images of dietary patterns, asking the user to select the one most like their current intake using a “this or that” process until “best possible fit” is achieved, similar to an eye exam. DietID can reliably determine dietary patterns within minutes with minimal participant burden even for those with cognitive decline. This is a key innovation in the approach of this study, which we hypothesized would support its feasibility. DietID outputs were used for descriptive dietary phenotyping, capturing relative consumption patterns and diversity rather than estimating absolute nutrient intake or adherence to a specific dietary prescription. This approach aligns with National Institutes of Health (NIH)/National Institute on Aging (NIA) guidance emphasizing pattern-based dietary characterization, particularly in aging and cognitive health research where feasibility, burden, and interpretability are key.

In recognition of the role of behavior change readiness in shaping dietary and lifestyle exposures, participants also completed a readiness-for-change survey. In short, this 32-question survey is a light adaptation of the University of Rhode Island Change Assessment, commonly referred to as the URICA.^31^ This instrument was designed to explore whether motivational stage and behavioral readiness were associated with gut microbiome features and cognitive outcomes. This item was made optional in an attempt to limit participant burden, and most participants opted in to the relatively short survey. The development of the survey is described in depth in a publication currently under review. In brief, the survey consists of a series of Likert-scale items (1 = strongly disagree to 5 = strongly agree) designed to capture motivational orientation toward dietary change, including denial or disengagement, contemplation, active behavior change, and maintenance or relapse prevention. Items reflect constructs aligned with the Transtheoretical Model of behavior change, including precontemplation, contemplation, action, and maintenance, as well as perceived usefulness of external tools (e.g., microbiome reports) to support behavior change. For analytic purposes, items were grouped conceptually according to stages of change (e.g., precontemplation, contemplation, action, maintenance/relapse prevention), consistent with prior readiness-to-change frameworks. Higher scores on action- and maintenance-oriented items reflect greater engagement in dietary behavior change, whereas higher endorsement of resistance-oriented items reflects lower readiness to change. Composite or subscale scores were derived by averaging relevant items, with higher scores indicating greater readiness to change nutritional habits.

Medical history, including medication and dietary supplement use, was recorded at each visit to support contextual interpretation of microbiome and clinical findings.

### Biospecimen collection and processing

Participants provided stool samples at each time point using standardized home collection kits. Samples were stored and processed following protocol-defined procedures to preserve microbial DNA integrity. Samples were banked for shotgun metagenomic analysis.

Participants self-collected fecal samples (≈0.25 g) at each study time point using standardized stool collection kits containing a nucleic acid preservation buffer optimized for gut microbiome analyses, as previously described.^32^ Following collection, samples were stored at ambient temperature and shipped to the processing laboratory within three days of collection. Upon receipt, all samples were logged into the laboratory information management system (LIMS) along with accompanying metadata and immediately stored at −80 °C until further processing to preserve microbial DNA integrity.

### Sample preparation, DNA extraction, shotgun metagenomics and metrology alignment

A microbial internal control was prepared using *Deinococcus radiodurans* (NIST 0032), *Brenneria nigrifluens* (NIST0080), and *Delftia acidovorans* (NIST 0136). Each organism was added to the CD1 lysis buffer (DNeasy PowerSoil Pro Kit, QIAGEN) at a concentration of 1.25 × 10^4^ cells/µL. Aliquots of 800 µL spiked buffer were prepared, resulting in 10^7^ cells per organism per aliquot. Participant stool samples were thawed at room temperature and homogenized via vortexing. A 250 µL volume of stool was transferred into the 800 µL spiked CD1 buffer, mixed briefly, and stored at −80 °C.

DNA extraction and shotgun metagenomic sequencing were performed by CosmosID (Germantown, MD; CLIA-certified, GCP compliant). Prepared stool samples (described above) were transferred to and stored at CosmosID at −80 °C until processing. Immediately prior to extraction, samples were thawed at room temperature then processed using the DNeasy PowerSoil Pro Kit following the manufacturer's instructions. Isolated DNA was quantified using the Qubit Flex fluorometer and the Qubit dsDNA HS Assay Kit (Thermo Fisher Scientific).

DNA libraries were prepared using the Watchmaker DNA Library Prep Kit (7K0019-1K). Genomic DNA was fragmented using a mastermix of Watchmaker Frag/AT Buffer and Frag/AT Enzyme Mix. IDT xGen UDI Primers and IDT Stubby Adapters were added to each sample followed by 7 cycles of PCR to construct the DNA libraries. The final DNA libraries were purified using CleanNGS magnetic beads (CleanNA) and eluted in nuclease-free water. Following elution, the libraries were quantified using the Qubit™ fluorometer dsDNA HS Assay Kit. Libraries were then circularized using the Element Adept library compatibility workflow and sequenced on the Element AVITI platform using the AVITI 2 × 150 Cloudbreak sequencing kit.

In addition to the internal controls, NIST RM 8048, a pooled human fecal reference material was included in the analysis. This NIST RM 8048 has been characterized extensively^33^ and provides a means of assessing workflow reproducibility.

### Statistical and bioinformatic analysis

Given the small sample size inherent to this feasibility study, analyses were limited to descriptive statistics and exploratory comparisons. Multivariable or covariate-adjusted models were not performed. Descriptive statistics characterized participant demographics and assessment scores. Microbial diversity metrics and relative taxonomic abundance were evaluated between cognitive groups. Findings are presented as hypothesis-generating rather than confirmatory. Functional pathway profiles analysis was not performed due to insufficient sample size.

Reads were first filtered using the fastp (v.0.23.4) program^34^ with the following parameters: a minimum quality Phred threshold set to 20, reads trimmed/truncated to bases 2–152, and the –detect_adapter_for_pe flag. Filtered reads were then aligned against a pre-masked, curated human reference genome (https://drive.google.com/file/d/0B3llHR93L14wd0pSSnFULUlhcUk/edit?usp=sharing) using bbmap (v.37.62; Bushnell, B. BBMap: A Fast, Accurate, Splice-Aware Aligner. (2014)) with the following recommended parameters: minid = 0.95, maxindel = 3, bwr = 0.16, bw = 12, quickmatch fast, minhits = 2, untrim. Unaligned reads were considered decontaminated and used as input for Kraken2 (v.2.1.2) taxonomic assignment^35^ with default parameters. The Kraken2 standard database was built on 11/21/2025.

We performed all statistical analyses in R using relative-abundance taxonomic profiles with samples annotated as healthy controls (HC), MCI, or RM8048 reference controls. Analyses were conducted at multiple taxonomic resolutions (phylum, genus, and species) after confirming the expected sample counts and metadata encoded in sample identifiers; replicate samples from the same individual were additionally grouped using a patient identifier parsed from the sample name. Although analyses were performed at all taxonomic levels, only phylum-level results are presented in this paper.

For community-level comparisons (beta diversity), we computed Bray–Curtis dissimilarities from abundance tables (samples × taxa) using the vegan package and visualized ordination structure with principal coordinates analysis (PCoA) using ape. Group-level structure was assessed visually using 95% confidence ellipses overlaid on PCoA plots (ggplot2). To test for global differences in community composition between HC and MCI, we used permutational multivariate analysis of variance (PERMANOVA) with adonis2 in vegan (9999 permutations). To evaluate whether any PERMANOVA findings could be influenced by differences in within-group dispersion, we performed multivariate dispersion testing (PERMDISP) using betadisper, visualized distances to group centroids with boxplots, and evaluated significance via permutation testing.

To assess whether group differences were driven by presence/absence rather than abundance, we additionally computed Jaccard distances by converting abundance tables to binary (taxon present if abundance > 0) and repeated PCoA visualization and PERMANOVA testing using the same framework.

Because microbiome abundance data are compositional and may contain zeros, we conducted sensitivity analyses evaluating zero handling. In addition to analyses on raw relative abundances (zeros retained), we performed multiplicative zero replacement using the zCompositions package (cmultRepl, CZM method) to generate zero-adjusted abundance tables. Bray–Curtis distances, PCoA visualizations, PERMANOVA, and dispersion testing were repeated on the zero-replaced data to confirm robustness of conclusions.

For univariate differential abundance screening, we compared taxa between HC and MCI using Wilcoxon rank-sum tests (implemented in base R) for each taxon at each resolution. *p*-values were adjusted for multiple comparisons using the Benjamini–Hochberg false discovery rate (FDR) procedure. Results were summarized with per-taxon box-and-whisker plots and, where helpful, stacked bar charts of taxonomic composition across samples (excluding “Unassigned” taxa when present) using ggplot2.

The study is reported in accordance with the reporting guidelines for human microbiome research: the Strengthening The Organization and Reporting of Microbiome Studies' (STORMS) checklist.

## Results

### Participant characteristics

Seventeen participants completed sufficient assessments and sample collection for inclusion in this analysis. Participants ranged in age from 54 to 86 y and represented HC and MCI—no early AD recruits were able to complete the requirements of a study visit. Therefore, our findings cannot be generalized across the full cognitive decline spectrum we intended. Attrition exaggerated uneven group sizes, with samples over representing healthy controls (*n* = 11) versus MCI (*n* = 6).

Given the small sample size, participant characteristics were treated descriptively and considered potential confounders rather than potentially explanatory variables (see Table 3). Age did not differ between healthy controls (66.5 y) and MCI (67.5 y). Fifteen were female with one male in each group. Eleven (66.7%) identified as Caucasian/White (8 HC, 3 MCI), five (27.8%) as African American/Black (3 HC, 2 MCI), and one (5.6%) as Asian (MCI). No differences in disease states stood out except for Irritable Bowel Syndrome (IBS) for which two of three (66.7%) were in the MCI group.

**Table 3.** Participant characteristics by cognitive group.

Characteristic	Healthy control (HC) (*n* = 11)	Mild cognitive impairment (MCI) (*n* = 6)	Total (*n* = 17)
Age, years, mean ± SD	66.5 ± 10.2	67.5 ± 7.9	66.8 ± 9.4
Female, *n* (%)	10 (91%)	5 (83%)	16 (89%)
Race/ethnicity, *n* (%)			
Caucasian/White	8 (73%)	3 (50%)	12 (67%)
African American/Black	3 (27%)	2 (33%)	5 (28%)
Asian	0 (0%)	1 (17%)	1 (6%)
Irritable bowel syndrome (IBS), *n* (%)	1 (9%)	2 (33%)	3 (17%)

Data are presented as mean ± standard deviation or number (percentage). The analytic cohort included healthy control and mild cognitive impairment participants only; no early Alzheimer's disease cases were represented in this subset. Given the small sample size, characteristics are reported descriptively.

Of the 17 participants, 14 completed the BoCA at baseline with 8 being HC (57.1%)—all MCI participants completed the BoCA at baseline (see Table 4). HC outperformed MCI on total BoCA score, with the largest group differences observed in visuospatial reasoning and overall cognitive performance. Several domains showed relative preservation across groups. HC exhibited a cognitive profile consistent with preserved multidomain function, supporting their classification and providing a stable reference group by this definition of cognitive health. The MCI group demonstrates a pattern consistent with early or mild impairment, characterized by selective domain vulnerability rather than global cognitive decline. These findings are presented to characterize cognitive profiles within the cohort and should not be interpreted as diagnostic confirmation.

**Table 4.** Baseline BoCA scores by cognitive status.

BoCA domain (max score)	HC mean	MCI mean	MCI–HC
Total score (30)	27.5	25.5	−2.0
Memory—immediate recall (2)	1.9	1.5	−0.4
Language/prefrontal synthesis (5)	4.5	4.5	0.0
Visuospatial reasoning (3)	2.8	1.5	−1.3
Executive function/clock (4)	3.6	3.8	0.2
Attention (4)	3.4	3.5	0.1
Mental Math (4)	3.8	3.3	−0.4
Orientation (3)	2.9	2.8	0.0
Memory—delayed recall (5)	4.8	4.5	−0.3

Values are mean scores; maximum score for each domain shown in parentheses. HC *n* = 8; MCI *n* = 6 at baseline. Differences are descriptive and not inferential.

At baseline, both HC and MCI participants reported relatively preserved physical function and low pain interference overall (Table 5). However, MCI participants reported greater symptom burden in anxiety, fatigue, and pain intensity, alongside reduced ability to participate in social roles and activities. HC participants exhibit a profile consistent with preserved functional and psychosocial well-being across PROMIS domains.

**Table 5.** Baseline PROMIS-29 domain scores by cognitive status.

PROMIS-29 domain	HC mean	MCI mean	MCI–HC
Physical function	4.8	4.5	−0.3
Anxiety	1.2	2.5	1.3
Fatigue	2.2	2.8	0.7
Sleep disturbance	1.9	2.1	0.2
Social roles & activities	4.7	3.5	−1.2
Pain interference	1.4	1.7	0.3
Pain intensity	1.0	2.7	1.7

Values represent mean domain scores averaged across items within each PROMIS domain. Directionality reflects original item scaling. Baseline visit only; HC and MCI classifications based on study criteria. Differences are descriptive only.

### Microbial taxonomy

**Figure 1.**
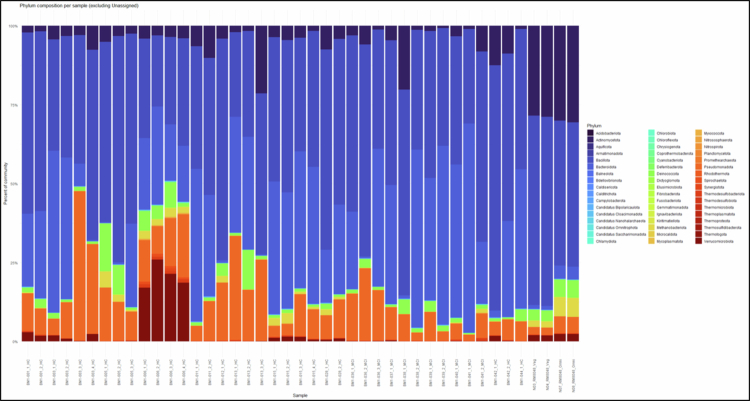
Gut microbiome composition at the phyla level. The barchart shows relative abundances (after removing unassigned reads) of all 51 phyla identified in stool samples. Each participant donated between 1 and 4 samples; and samples from the same donor have the same 4-digit identifier. HC and MCI denote healthy controls and mild cognitive impairment, respectively. NIST RM 8048 vegetarian (veg) and omnivore (omni) fecal reference materials were run in duplicate (last four columns).

**Figure 2.**
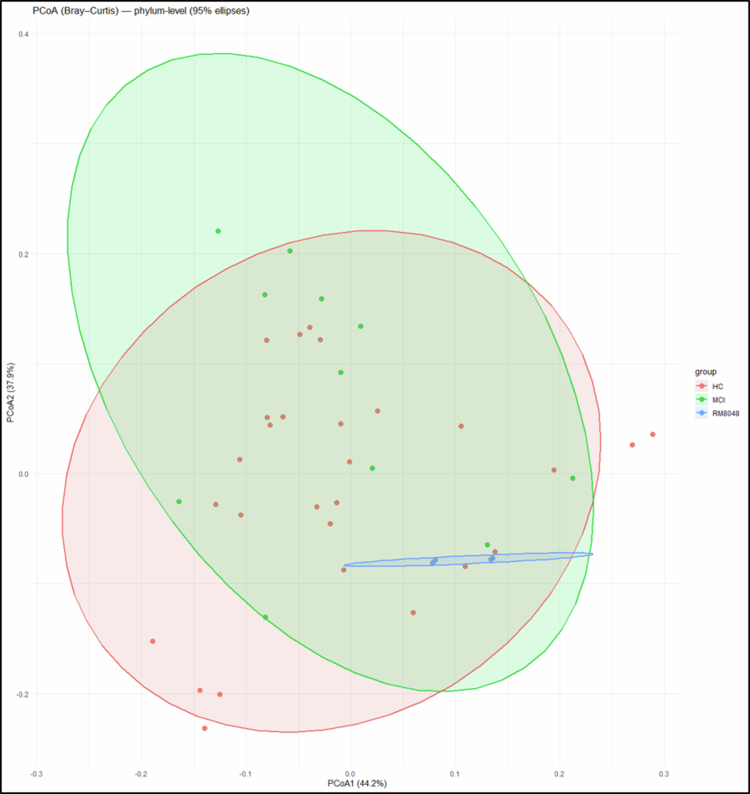
Gut microbiome composition PCoA (Bray–Curtis With Confidence Ellipses) by cognitive status. Principal coordinates analysis (PCoA) visualization using Bray–Curtis dissimilarity matrices generated from gut microbiome compositions between healthy controls (HC) in orange, mild cognitive impairment (MCI) in green, and NIST RM 8048 in blue. Ellipses encompass 95% of each group's data.

Microbial taxonomy was assessed at the Phylum level. Fifty-one (51) unique phyla were identified across all samples (*n* = 43), including the NIST reference material, RM 8048; however, most samples were dominated by approximately 10 phyla (Figure 1). Across all samples the 10 most common phyla were: Bacillota, Bacteroidota, Pseudomondota, Actinomycetota, Deinococcota, Verrucomicrobiota, Methanobacteriota, Thermodesulfobacteriota, Camplyobacterota, and Spirochaetota. Of note, Deinococcota is the phylum to which the one of the organisms that was spiked in to the samples as an internal control is assigned. Surprisingly, no overt differences between the two populations were observed (Figure 1). This was further supported by PCoA visualization wherein substantial overlap was observed between the HC and MCI group samples' distributions (Figure 2). RM 8048 Vegetarian and RM 8048 Omnivore internal control samples (*n* = 2) were also substantially covered by the HC and MCI distribution ellipses (Figure 2). Similar analyses were also conducted at higher taxonomic resolutions (e.g. genus, species) with similar results (data not shown).

### Diet quality

The DietID assessment was completed at baseline by 15 of 17 participants; the summary results are described in Table 6. This subset was slightly younger than the total group (64.9 vs. 66.8 y), as the eldest participant (86 y) did not complete it. The other missing participant was one of the two youngest (54 y). The average participant was overweight by BMI category. The most common dietary pattern was Flexitarian.

**Table 6.** Dietary intake assessment by cognitive status. **Intense**: engage in very intense exercise, sports or a physical occupation.

			HC (*n* = 9)		MCI (*n* = 6)		Total (*n* = 15)	
Female (*n*, %)			8	88.9%	5	83.3%	13	86.7%
Age (y)			63.9	±8.6	67.2	±8.1	65.1	±8.3
Weight (lbs)			155.8	±43.3	147.3	±26.1	152	±36.5
Height (in)			64.6	±3.4	64	±2.6	64.3	±3.0
BMI			26.8	±9.4	25.2	±3.7	26.1	±7.5
**Dietary pattern (*n*, %)**								
Flexitarian			2	22.2%	2	33.3%	4	26.7%
Low-carb			1	11.1%	1	16.7%	2	13.3%
Low-fat			1	11.1%	1	16.7%	2	13.3%
Mediterranean			0	0.0%	1	16.7%	1	6.7%
No Red meat			1	11.1%	1	16.7%	2	13.3%
Paleo			2	22.2%	0	0.0%	2	13.3%
Pescatarian			1	11.1%	0	0.0%	1	6.7%
Vegan			1	11.1%	0	0.0%	1	6.7%
**Dietary quality (average)**								
DietID index			8.0		8.0		8.0	
Healthy eating index (HEI)			81.6	±6.9	80.3	±7.6	81.0	±7.2
**Dietary components (servings/d)**								
Vegetables			8.1	±2.9	5.5	±2.3	6.9	±3.0
Fruit			3.0	±0.9	2.0	±0.8	2.6	±0.9
Whole grains			2.4	±1.4	2.9	±1.3	2.6	±1.3
Nuts and seeds			1.9	±1.0	1.3	±0.8	1.6	±1.0
Fish			2.7	±1.3	1.5	±1.1	2.1	±1.3
Poultry			1.1	±0.6	0.7	±0.5	0.9	±0.6
Red and processed meat			1.5	±0.9	1.6	±1.0	1.5	±0.9
Animal protein composite			0.6	±0.5	0.7	±0.5	0.7	±0.5
Unsaturated fats			4.3	±2.3	3.7	±2.4	4.1	±2.4
Unsweetened beverages			2.4	±0.9	2.2	±1.0	2.3	±0.9
Sweetened beverages			0.4	±0.5	0.0	±0.0	0.2	±0.4
Sweets and desserts			0.4	±0.5	1.1	±0.8	0.7	±0.8
Sweet-salty condiments			0.7	±0.6	1.2	±0.7	0.9	±0.7
**Activity level (*n*, %)**								
Intense			1	11.1%	0	0.0%	1	6.7%
Active			2	22.2%	1	16.7%	3	20.0%
Moderate			2	22.2%	1	16.7%	3	20.0%
Light			3	33.3%	2	33.3%	5	33.3%
Minimal			1	11.1%	2	33.3%	3	20.0%
**Do you typically choose USDA-certified organic foods? (*n*, %)**								
Yes			1	11.1%	2	33.3%	3	20.0%
Often			1	11.1%	0	0.0%	1	6.7%
Sometimes			1	11.1%	0	0.0%	1	6.7%
Milk only			1	11.1%	0	0.0%	1	6.7%
Rarely			1	11.1%	0	0.0%	1	6.7%
No			1	11.1%	0	0.0%	1	6.7%
No response			3	33.3%	4	66.7%	7	46.7%

### Dietary patterns

**Flexitarian** – mostly vegetarian diet that sometimes includes meat, fish, and/or poultry. May include highly processed foods, beverages, and ingredients.

**Low-carb** – includes lean meats, poultry, seafood, eggs, mostly non-starchy vegetables, whole fruits, nuts and seeds, variety of fats, with or without dairy/non-dairy products. Limits grains, legumes, and added sugars.

**Low-fat** – includes lean meats, poultry and fish, fruits and vegetables, grains, legumes, and low-fat dairy products. May include low-fat processed foods and limited amounts of nuts, seeds, nut butters, olives, avocado, cooking oils, fatty fish, and eggs.

**Mediterranean** – includes vegetables, fruits, nuts and seeds, whole grains, legumes, dairy products, seafood and lean poultry. Emphasis on olive oil, herbs, spices, and optional red wine (moderation).

**No red meat** – includes fruits, vegetables, grains, beans, nuts, seeds, dairy products, eggs, poultry, and fish. May include highly processed foods, beverages, and ingredients.

**Paleo** – includes a variety of meats (e.g. grass-fed, game), wild fish and seafood, free-range eggs, whole fruits and vegetables, nuts and seeds, with or without non-dairy milk. Limits legumes, grains, dairy products, refined sugars and added salt.

**Pescatarian** – includes seafood (fin fish and shellfish), vegetables, fruits, grains, legumes, nuts, seeds, dairy products, and eggs. May include highly processed dairy and plant-based foods. Excludes all other animal products.

**Vegan** – comprised of 100% plant-based foods including vegetables, fruits, grains, legumes, nuts, and seeds, as well as processed foods such as baked goods, soyfoods, oils, dairy substitutes, meat substitutes, and sweets/desserts made without dairy or egg. Excludes all animal products.

Diet quality

**Table:** 

DietID index	HEI score
10	96 to 100
9	90 to 95
8	80 to 89
7	70 to 79
6	60 to 69
5	50 to 59
4	40 to 49
3	30 to 39
2	20 to 29
1	<20

**Activity level.**

**Minimal**: little or no exercise.

**Light**: engage in exercise or sport activities 1 times/week–3 times/week.

**Moderate**: engage in exercise or sports activities 3 times/week–5 times/week.

**Active**: engage in exercise or sport activities 6 times/week–7 times/week.

Diet quality was higher than the average American with Healthy Eating Index 2020 (HEI-2020) of 81 versus 58 in National Health and Nutrition Examination Survey (NHANES).^36^ ^,^ ^37^ While almost half did not answer the free-text question: “Do you typically choose USDA-certified Organic foods?”, most respondents chose United States Department of Agriculture (USDA)-certified organic foods at least some of the time. Organic consumption has become mainstream, as an estimated 80% of U.S. households purchase organic products, to varying degrees.^38^

Physical activity levels varied across both cognitive groups, with a greater proportion of MCI participants reporting minimal activity (little or no exercise).

Across participants, DietID revealed substantial interindividual variability in dietary patterns, particularly in the intake of vegetables, whole grains, unsweetened beverages, and fat quality. No single dominant dietary pattern characterized the cohort, supporting the use of diet as a heterogeneous exposure rather than a uniform background factor. The following is a summary of the dietary patterns with estimates of daily intake by servings:

- Vegetables
  ∘ Mean: 7.0 ± 3.1, Range: 2.8 to 10.6
  ∘ Vegetable intake ranged widely, from very low to relatively high, indicating meaningful variation in plant-forward eating.
  ∘ Intake of vegetable juice was negligible across participants, suggesting that vegetable exposure was largely food-based rather than beverage-driven.
- Whole Grains
  ∘ Mean: 3.6 ± 2.7, Range: 0 to 6.8
  ∘ Whole grain intake similarly showed broad dispersion, with some participants reporting minimal exposure and others reporting moderate-to-high intake.
- Fat Quality: Unsaturated Fats
  ∘ Mean: 4.0 ± 2.4, Range: 0 to 5.8
  ∘ Intake of unsaturated, healthier fats varied considerably across participants.
  ∘ Some participants showed patterns consistent with regular inclusion of plant oils, nuts, seeds, or fatty fish, while others showed minimal exposure.
- Beverages: Unsweetened
  ∘ Mean: 2.3 ± 0.93, Range: 0.98 to 3.2
  ∘ Unsweetened beverage intake (e.g., water, tea, coffee without added sugar) varied substantially.
- Animal Protein and Mixed Dietary Patterns
  ∘ Mean: 0.63 ± 0.5, Range: 0 to 1.6
  ∘ Composite of Red meat, Processed meat, Poultry, Fish, Shellfish, Eggs, Full-fat dairy products, and Reduced- or non-fat dairy products.
  ∘ Screening responses indicate mixed dietary patterns, with participants variably consuming meat, poultry, and fish.
  ∘ No strict vegetarian or vegan pattern dominated the cohort.

Compared with HC, the MCI group showed lower fish, fruit, nuts/seeds, and plant-based dairy alternatives, and higher sweets/desserts and sweet-salty condiments, with similar-to-slightly higher whole grains. DietID data demonstrated substantial heterogeneity in dietary patterns across participants, particularly in vegetable intake, whole grains, fat quality, and unsweetened beverage consumption. These findings are presented to characterize dietary exposures within the cohort and to support dietary phenotyping, rather than to assess dietary adequacy or adherence to a specific dietary pattern.

### Nutrition behavior readiness to change

Only 9 of 17 participants opted into the optional Readiness to Change Survey with age ranging from 54 to 76 (average: 65.2) with more being MCI (6, 66.7%) and all MCI participants completing this element (as with the BoCA), see Table 7. Overall, participants demonstrated high readiness to engage in nutritional behavior change, with responses clustering strongly in the contemplation, action, and maintenance domains and very limited endorsement of precontemplation or resistance-oriented statements. Items reflecting thinking about change, recognition of room for improvement, and perceived value in working on nutritional habits were rated highly across participants. Nearly all participants agreed or strongly agreed that improving nutrition would be worthwhile and that they had been contemplating changes. Statements indicating active steps toward improving nutritional habits were among the most strongly endorsed items. Participants frequently agreed that they were already working on their nutrition, taking action, and making meaningful changes rather than merely discussing them. Despite strong action-oriented responses, many participants also endorsed statements reflecting concern about relapse, difficulty maintaining changes independently, or desire for additional ideas and support. These patterns indicate that while participants are motivated and engaged, sustained behavior change remains a challenge.

Both HC and MCI participants demonstrated high readiness to engage in nutritional behavior change, with strong endorsement of contemplation and action-oriented items (Table 7). However, qualitative differences emerged in maintenance confidence and relapse concern. HC participants appear to be in late action to early maintenance, with relatively high self-efficacy. MCI participants appear more concentrated in action-with-support and maintenance-with-risk-of-relapse, highlighting the importance of scaffolding and sustained guidance in this group.

**Table 7.** Readiness to change survey by cognitive status.

Survey domain (representative items)	HC mean	MCI mean	MCI–HC
No need to change/denial	2.3	2.3	0.0
Ready for improvement	4.3	4.2	−0.2
Actively taking steps	5.0	3.7	−1.3
Working hard to change	5.0	3.7	−1.3
Concern about maintaining changes	2.0	3.8	1.8
Relapse or recurrence concerns	2.3	3.2	0.8
Desire for help/support	4.0	4.5	0.5
Openness to microbiome report	4.7	5.0	0.3
Microbiome report seen as waste	1.0	1.7	0.7
Boredom or resistance to nutrition	1.3	2.0	0.7

Values are mean Likert scores (1–5). Positive values in the final column indicate higher endorsement among MCI participants. HC *n* = 3; MCI *n* = 6. Differences are descriptive and not inferential.

## Discussion

This proof-of-concept study demonstrates the feasibility of integrating longitudinal cognitive and behavioral phenotyping with shotgun metagenomics in a cognitively diverse adult cohort. In alignment with our recent scoping review, these findings underscore the substantial overlap in microbiome characteristics between HC and MCI and the difficulty of detecting group-level differences in small, heterogeneous cohorts without sufficient statistical power or representation across the full cognitive spectrum, including early AD.^16^ Given the high interindividual variability of the human gut microbiome, cohorts of this size are unlikely to detect subtle disease-associated microbial signals. The absence of significant group-level microbiome differences should not be interpreted as a failure to detect meaningful biology. Rather, in early cognitive impairment, substantial biological overlap is expected, and such findings underscore the limitations of cross-sectional, taxon-centric inference in this disease stage, especially in small cohorts such as this.

The BoCA offers several methodological advantages for longitudinal cognitive monitoring in research settings. Prior validation studies have demonstrated strong psychometric properties, including high internal consistency, excellent test-retest reliability, and robust convergent validity with established cognitive screening measures such as the Montreal Cognitive Assessment (MoCA) and the Telephone Interview for Cognitive Status (TICS). A distinctive feature of the BoCA is its use of randomly selected, non-repeating tasks, which minimizes practice effects and permits repeated administration without learning bias. This is an important innovation in the approach of this study, supporting its feasibility.

This study highlights several challenges in the quest for microbiome signatures associated with cognitive state. First, despite selecting a commonly used bioinformatics analysis workflow, a substantial proportion of the sequencing reads (33%–55%) remained unassigned even at the domain level. This observation likely reflects a combination of factors, including reference database selection, limitations and biases associated with current taxonomic classification algorithms, and the presence of poorly characterized microbial taxa in our samples, which is not a novel finding. Importantly, such uncertainty constrains taxonomic resolution and may obscure potentially meaningful biological differences between groups, particularly small cohorts such as this. The proportion of unassigned reads is often overlooked in microbiome studies, for example, some tools will drop the unassigned reads during analysis and only present the relative abundance data based on the taxonomically classified reads. It is critical for microbiome studies to report the algorithm-calculated proportions of unassigned reads considering the implications associated with comparisons between microbiomes with different microbial compositions and putative identifications of disease-associated taxa. Additional analyses are underway to further investigate the causes of unassigned reads as well as to improve taxonomic assignment where possible. High proportions of unassigned reads highlight the need for expanded reference databases and measurement-informed analytical frameworks.

Although shotgun metagenomic sequencing was performed, analyses in the present study were limited to taxonomic composition and small sample size. Therefore, functional pathways analysis was not conducted. This represents an important limitation, as microbiome-brain interactions are likely mediated through microbial metabolic products, immune signaling pathways, and host-microbe interactions rather than taxonomic composition alone. As such, reliance on taxonomic profiles may obscure functionally relevant differences between groups. Future studies should prioritize integration of functional pathway analysis, metagenomic inference of microbial metabolic capacity, and complementary approaches such as metabolomics and host immune profiling to better characterize the mechanistic links between the gut microbiome and cognitive health. These approaches will be particularly important when combined with metrology-informed workflows to improve reproducibility and cross-study comparability.

Another challenge is the biological heterogeneity of the samples. Notably, the distributions of samples associated with each group largely overlapped each other despite the presence of cases wherein multiple stool samples were donated from a single donor. This phenomenon highlights the biological variability in stool composition from a single individual, thus, raising the minimum threshold required to observe major differences between different individuals and groups. Moreover, the internal control samples (i.e., RM 8048 Vegetarian and Omnivore) were completely within the distributions of both the HC and MCI cohorts' microbiomes which further illustrated the inability to differentiate the two cohorts. In the absence of overt compositional differences between groups, additional techniques will be needed to tease out cohort-specific associations with the biological heterogeneity.

Contributing to this challenge is a central limitation of microbiome sequencing data analysis, namely microbial compositionality among different samples. In metagenomic sequencing, reported taxon abundances are typically relative, meaning that an apparent change in one organism can induce spurious changes in others because all values are constrained to a fixed total. This can complicate interpretation, reduce comparability across samples and studies, and obscure true biological differences, particularly in heterogeneous human cohorts. A recent NIST-led study formalized this measurement problem and introduced a mathematical framework using exogenous internal standards to correct compositional distortions and derive scaled abundances that more closely reflect underlying biological abundance.^39^ Internal standards were included in this sample set, and follow-up analyses are underway to apply this framework to this study.

There were two additional complicating factors that emerged through the course of this study, first several healthy controls reported motivation to participate in this study due to family history; it begs the question if these healthy controls are not, in fact, somewhere on the cognitive decline spectrum in that they may be at-risk. Specifically, this is supported by the fact that Pseudomonadota, while common at low relative abundance, is seen at greater abundance in dysbiosis, inflammation, or disease states. Individual species analysis supports this; though, none of this reaches statistical significance due to insufficient power.

Second, this study highlights the difficulty of recruiting individuals and/or caregivers to research cognitive decline—a population already under substantial burden. Despite attempts to reduce participant burden (e.g. decentralized model not requiring in-person visits and travel, at-home stool sample collection with an easy-to-use kit, targeted questionnaires with limited questions, rapid dietary assessment method) recruitment of individuals with early AD was extremely difficult—only mildly less so for MCI.

After recruitment, stool sample collection was a significant hurdle with many participants requiring replacement kits or ultimately declining to send samples after having difficulty, especially in the early AD and MCI arms. Newer collection approaches (i.e. Bunny Wipe vs. scoop collection) may reduce the aversion (“ick factor”) and difficulty for sample collection in those with cognitive decline and their caregivers. Caregiver involvement when a participant is not sufficiently independent is a larger barrier in many ways—making even early AD a major barrier to recruitment. Promising technology for improving sample collection in this population also includes the BiomeSense GutLab™ (Chicago, IL), which is placed by the toilet and toilet paper deposited after wiping. This is a significant improvement on the ease of sample collection, greatly reducing the ick factor. Such advances may be the key to recruiting robust longitudinal cohorts to definitively study the role of the gut microbiome and lifestyle in cognitive decline; however, currently, the cost of such devices are beyond the budgets typical of exploratory clinical trials.

The literature on the role of diet in modifying the composition and function of the gut microbiome continues to grow and consistently identifies diet as a central, modifiable determinant.^40-43^ Dietary assessment in this cohort revealed substantial interindividual variability in habitual intake, supporting treatment of diet as a heterogeneous exposure rather than a uniform background factor. Although overall diet quality exceeds U.S. population averages, participants did not consistently adhere to a single dietary pattern previously associated with cognitive resilience, such as Mediterranean or MIND-style diets. The most common dietary pattern was flexitarian, with wide dispersion in intake of vegetables, whole grains, unsaturated fats, and fish—dietary components known to influence gut microbial diversity and metabolic output. This heterogeneity likely contributed to the variability in microbiome composition and functional capacity across participants and highlights the importance of contextualizing microbiome findings within habitual dietary exposure rather than assuming diet as static or controlled. Further, the substantial interindividual variability in habitual dietary intake observed in this cohort likely contributed to the overlap in microbiome composition between cognitive groups. In the absence of dietary standardization or stratification, diet may act as a dominant, heterogeneous exposure that masks subtle disease-associated microbial signals, particularly in early cognitive decline.

In addition to dietary heterogeneity, several other factors known to influence gut microbiome composition were present in this cohort and may have contributed to interindividual variability. These include medication use, dietary supplements, comorbid conditions, adiposity, and physical activity, all of which have been shown to shape microbial composition and function. Although these variables were recorded to support contextual interpretation, the small sample size precluded stratified or multivariable analyses. As a result, these factors may have influenced observed microbial profiles and further obscured potential group-level differences between healthy control and MCI, reflecting both real-world complexity and a source of analytic limitation in small cohorts.

At baseline, participants with MCI demonstrated lower overall BoCA scores than cognitively healthy participants, with the largest differences observed in visuospatial reasoning, while language, executive function, attention, and orientation remained largely preserved. Importantly, BoCA findings are interpreted here as phenotypic descriptors of cognitive function, not as diagnostic markers, consistent with the study's emphasis on multidimensional characterization.

According to PROMIS-29, participants with MCI reported greater anxiety, fatigue, pain intensity, and reduced social participation despite largely preserved physical function. These findings highlight that individuals with MCI may experience greater emotional, social, and symptom burden even in the absence of marked physical impairment, reinforcing the value of integrative interventions that address psychological well-being, fatigue, pain perception, and social engagement alongside cognitive and nutritional strategies.

Participants generally rejected statements suggesting that nutrition was not relevant or that change was unnecessary, indicating broad recognition of the role of nutrition in health. Overall readiness-for-change profiles suggest that participants were beyond precontemplation and largely engaged in contemplative or action-oriented stages. Items assessing perceptions of the microbiome report were rated favorably, with participants expressing interest in receiving personalized, data-informed feedback. Both healthy control and MCI participants demonstrated high motivation for nutritional change; however, individuals with MCI reported greater concern about maintaining changes and a stronger desire for external support. These patterns suggest that while nutrition-focused interventions are likely to be well received across cognitive groups, individuals with MCI may benefit from simplified guidance, reinforcement, and ongoing accountability.

The combined use of cognitive screening, patient-reported outcomes, dietary assessment, and behavioral readiness measures reflects a phenotyping-first approach aligned with NIH/NIA priorities for understanding heterogeneity in cognitive aging. Preservation of executive function and physical capacity alongside increased anxiety, fatigue, and social participation challenges among participants with MCI highlights the importance of moving beyond diagnosis alone to characterize intervention-relevant functional profiles. This approach supports the development of tailored, scalable interventions that address cognitive health in the context of lived experience, consistent with NIA's emphasis on translational relevance and person-centered outcomes.

These findings should be interpreted in the context of several methodological and design limitations, summarized in the Limitations section below.

### Strengths

This study has several notable strengths that support its contribution as a feasibility and methods-forward investigation in an evolving field.

First, the study employed a multidimensional, phenotyping-first design, integrating cognitive assessment, patient-reported outcomes, dietary intake, behavioral readiness for change, and gut microbiome profiling within a single longitudinal framework. This whole-person approach aligns with NIH and NIA priorities emphasizing heterogeneity, lived experience, and functional relevance in cognitive aging research. Rather than relying on microbiome data in isolation, the study intentionally contextualized biological findings within behavioral, psychosocial, and lifestyle domains that are increasingly recognized as integral to brain health and intervention feasibility.

Second, the use of shotgun metagenomic sequencing, rather than 16S rRNA sequencing, enabled higher-resolution characterization of the gut microbiome and avoided many of the taxonomic and functional limitations inherent to marker-gene approaches. Although the study was not powered for functional pathway analyses, the sequencing strategy establishes a foundation for future work that can integrate taxonomic, functional, and metabolic inference in a more biologically meaningful way.

Third, this study incorporated metrology-aligned practices, including the use of exogenous microbial internal standards and NIST human fecal reference material (RM 8048). This measurement-centered framework directly addresses one of the most persistent challenges in human microbiome research: reproducibility and comparability across studies.

Fourth, the decentralized study model represents a practical strength, particularly for populations experiencing cognitive impairment. At-home stool collection, remote assessments, and brief, validated tools reduced participant burden and increased accessibility. Importantly, the successful implementation of this model demonstrates that longitudinal, multimodal data collection is feasible even in cognitively vulnerable populations, informing the design of future larger-scale studies.

Fifth, the study leveraged validated and innovative assessment tools selected specifically for feasibility and longitudinal use in aging populations. BoCA minimized practice effects, enabling repeated administration without learning bias. DietID provided rapid, low-burden dietary phenotyping without reliance on recall or detailed logging, which can be challenging for individuals with cognitive decline. The inclusion of a readiness-for-change assessment further strengthened the translational relevance by capturing motivational context often absent from microbiome and nutrition studies.

Finally, the study achieved meaningful racial diversity, including successful recruitment of African American/Black participants, a group historically underrepresented in cognitive aging and microbiome research. Collaboration with a diverse Memory Clinic and use of a decentralized design likely contributed to improved accessibility and participation, strengthening the relevance of the findings and providing important insights for equitable recruitment strategies.

### Limitations

Several limitations should be considered when interpreting these findings.

Foremost, the small sample size and resultant lack of statistical power limit the ability to detect group-level differences or draw inferential conclusions. The study was intentionally designed as a proof-of-concept and feasibility investigation, and all analyses are exploratory and descriptive. Attrition further contributed to imbalanced group sizes, particularly the absence of early Alzheimer's disease participants in the analytic sample, which constrained the ability to examine microbiome features across the full cognitive decline spectrum originally envisioned.

Second, the observational design precludes causal inference. While longitudinal data collection was implemented, the study was not designed to test mechanistic pathways or intervention effects. Associations described herein should therefore be interpreted as hypothesis-generating rather than explanatory.

Third, although validated cognitive and patient-reported instruments were used, the study did not include biomarker-based diagnostic confirmation (e.g., amyloid or tau imaging, cerebrospinal fluid markers). Cognitive status classifications relied on pre-existing clinical diagnoses and standardized assessments, which were intentionally used for phenotypic characterization rather than diagnostic adjudication. As such, misclassification remains possible, particularly among healthy control participants.

Relatedly, many healthy control participants reported enrolling due to a family history of cognitive decline, suggesting that this group may represent an at-risk or pre-clinical population rather than a truly unaffected reference group. This recruitment pattern may introduce self-selection/volunteer bias and reduce biological contract between groups, particularly if some individuals classified as cognitively healthy are on a trajectory toward cognitive decline (prodromal). Such overlap may contribute to the observed similarity in microbiome composition between healthy control and MCI. This likely reduced contrast between groups and further limited the ability to detect microbiome differences, particularly in a small cohort such as this. Future studies may benefit from more clearly defined control groups or from explicitly characterizing at-risk individuals as a separate arm rather than including them in the control group because they are currently considered cognitively normal. This highlights the importance of precise phenotyping and cohort definition in microbiome studies of cognitive aging.

Fourth, microbiome data interpretation was constrained by compositionality, dietary heterogeneity, and database limitations. A substantial proportion of sequencing reads remained unassigned, reflecting incomplete reference databases and the presence of poorly characterized taxa. While internal standards were included to support future compositional correction, scaled abundance analyses were not yet applied in the current dataset. These limitations underscore ongoing challenges in species-level inference and reinforce the need for measurement-informed analytical frameworks.

Finally, participant burden related to stool collection emerged as a significant barrier, particularly among individuals with MCI and early Alzheimer's disease. Despite use of standardized at-home kits and a decentralized model, stool collection proved to be a major point of attrition. This highlights the need for alternative collection methods and caregiver-supported approaches in future studies targeting cognitively impaired populations.

### Future studies

This study included the well-characterized RM 8048 human fecal reference standards to begin a measurement-centered framework emphasizing accuracy, traceability, and analytical consistency. Continuing from this exploratory work, these standards will be utilized at a higher degree in future work to advance microbiome science from discovery to application (i.e., translational microbiome sciences).

Based on the exploratory findings discussed here, future work will need to prioritize larger, more diverse cohorts with longitudinal designs and extended follow-ups to increase statistical power, improve findings generalizability, and better elucidate attrition introduced imbalance across cognitive groups in a temporal microbiome-associated framework. Additionally, standardized metagenomic workflows and integration of additional omics layers (e.g., metabolomics and host biomarkers) will need to be implemented.

## Conclusions

This study establishes the practical feasibility of conducting a decentralized, multidimensional investigation of the gut microbiome in adults with early cognitive decline using metrology-aligned shotgun metagenomics. By integrating cognitive assessment, dietary and behavioral phenotyping, and standardized microbiome analysis, the bMicrobiome Study demonstrates that comprehensive data collection in this population is achievable, even in the context of recruitment and retention challenges.

The findings underscore the substantial biological and behavioral heterogeneity present among individuals classified as cognitively healthy or with mild cognitive impairment. Rather than identifying discrete microbial signatures that differentiate cognitive groups, this work highlights the limitations of species-level inference in small, exploratory cohorts and reinforces the importance of measurement rigor, functional context, and cautious interpretation, especially when evaluating low-abundance microbiome signals. The prominence of poorly characterized taxa further illustrates the extent of microbial “dark matter” in human datasets and the need for improved reference frameworks.

Importantly, the study situates gut microbiome features within a broader behavioral and lifestyle context. High readiness for nutritional change and variability in habitual dietary patterns emphasize that diet and behavior are not static background variables but integral components of gut–brain research that may shape both microbiome composition and the feasibility of future interventions. These insights are methodological rather than prescriptive and are intended to inform study design rather than clinical decision-making.

Taken together, this work supports a phenotyping-first, measurement-informed approach to studying the gut microbiome in cognitive aging. While the results are exploratory and not generalizable, they provide a foundation for larger, longitudinal investigations that can more definitively evaluate temporal dynamics, functional relevance, and the potential role of integrative, nutrition-focused strategies earlier in the cognitive decline trajectory.

## Data Availability

De-identified data supporting the findings of this study are available from the corresponding author upon reasonable request, subject to institutional and data use approvals. Sequencing data are available on NCBI (BioProject: pending). to: “Sequencing data have been deposited in the National Center for Biotechnology Information (NCBI) under BioProject accession PRJNA1475489. Associated BioSample accessions SAMN60643521–SAMN60643559 are available through the BioProject record”.

## Disclaimer

Certain equipment, instruments, software, or materials are identified in this paper in order to specify the experimental procedure adequately. Such identification is not intended to imply recommendation or endorsement of any product or service by NIST, nor is it intended to imply that the materials or equipment identified are necessarily the best available for the purpose.
